# Mechanism of Cordyceps Cicadae in Treating Diabetic Nephropathy Based on Network Pharmacology and Molecular Docking Analysis

**DOI:** 10.1155/2021/5477941

**Published:** 2021-09-28

**Authors:** Yi Qian, Xin Sun, Xin Wang, Xin Yang, Mengyao Fan, Jiao Zhong, Zejun Pei, Junping Guo

**Affiliations:** ^1^Department of Pharmacy, The Affiliated Wuxi No. 2 People's Hospital of Nanjing Medical University, Wuxi 214002, China; ^2^Yixing People's Hospital, Yixing 214200, China

## Abstract

**Objective:**

To systematically study the mechanism of cordyceps cicadae in the treatment of diabetic nephropathy (DN) with the method of network pharmacology and molecular docking analysis, so as to provide theoretical basis for the development of new drugs for the treatment of DN.

**Methods:**

TCMSP, Symmap, PubChem, PubMed, and CTD database were used to predict and screen the active components and therapeutic targets for DN. The network of active components and targets was drawn by Cytoscape 3.6.0, the protein-protein interaction (PPI) was analyzed by the STRING database, and the DAVID database was used for the enrichment analysis of intersection targets. Molecular docking studies were finished by Discovery Studio 3.5.

**Results:**

A total of 36 active compounds, including myriocin, guanosine, and inosine, and 378 potential targets of cordyceps cicadae were obtained. PPI network analysis showed that AKT1, MAPK8, and TP53 and other targets were related to both cordyceps cicadae and DN. GO and KEGG pathway analysis showed that these targets were mostly involved in R-HSA-450341, 157.14-3-3 cell cycle, and PDGF pathways. Docking studies suggested that myriocin can fit in the binding pocket of two target proteins (AKT1 and MAPK8).

**Conclusion:**

Active ingredients of cordyceps cicadae such as myriocin may act on DN through different targets such as AKT1, MAPK8, and TP53 and other targets, which can help to develop innovative drugs for effective treatment of DN.

## 1. Introduction

Diabetic nephropathy (DN), also known as diabetic kidney disease (DKD), is one of the most common secondary nephrosis with high incidence rate and low cure rate. DKD develops in approximately 40% of patients who are diabetic and is the leading cause of chronic kidney disease (CKD) worldwide [1]. It increases the death rate in diabetic patients [2]. At present, there are effective approaches that can reduce the incidence of diabetic kidney disease and postpone its progression such as controlling blood glucose levels and blood pressure as well as blockade of the renin-angiotensin-aldosterone system [3]. Many researches of traditional Chinese medicine have also been carried out in the treatment of DN and have made some achievement in recent years, such as berberine [4], Qidan Dihuang grain (QDDHG) [5], and Danggui Shaoyao San (DSS) [6]. The mechanism of action may be multifaceted, such as regulating glucose metabolism, correcting lipid metabolism disorder, inhibiting the activation of polyol pathway, antioxidative stress, improving the structure and function of podocytes, inhibiting inflammatory response, and intervening cell signal transduction. However, there remains an urgent need for innovative drugs to treat DN.

Cordyceps cicadae, also called “Chan Hua,” belongs to the family Clavicipitaceae, Ascomycotina, and its anamorph is Isaria cicadae Miq. Furthermore, cordyceps cicadae has been used as a substitute for cordyceps sinensis [7]. Cordyceps cicadae is one of the most famous traditional Chinese medicines and has been used for about 1600 years in China. Cordyceps cicadae is a kind of cordyceps fungus produced by Paecilomyces sp. after infecting cicada. It belongs to the same insect fungus complex as cordyceps sinensis. Li's research has suggested that HEA, one active component of cordyceps cicadae, could alleviate many diabetes complications in genetically obese mice and may offer promise as a supplement for diabetes management [8]. Another study showed that cordyceps cicadae had the antidiabetic activity in a diabetic rat model and could be a promising therapeutic source in managing diabetes mellitus and its associated complications [9]. It was reported that the results indicated that CCP, one important component of cordyceps cicadae, improved insulin resistance and glucose tolerance in DN rats. Furthermore, CCP intervention significantly suppressed the inflammation, renal pathological changes, and renal dysfunction, slowing down the progression of renal interstitial fibrosis [10].

Network pharmacology, which is aimed at studying the complex, diverse relationships between targets, drugs, diseases, and pathways, presents a new approach for drug discovery [11]. Network pharmacology explores the relationship between traditional Chinese medicine and disease at the overall level, which provides a simple way to explain the mechanism of traditional Chinese medicine treatment. Network pharmacology uses bioinformatics to help us get a better understanding of drug actions and thereby to advance drug discovery.

**Figure 1:**
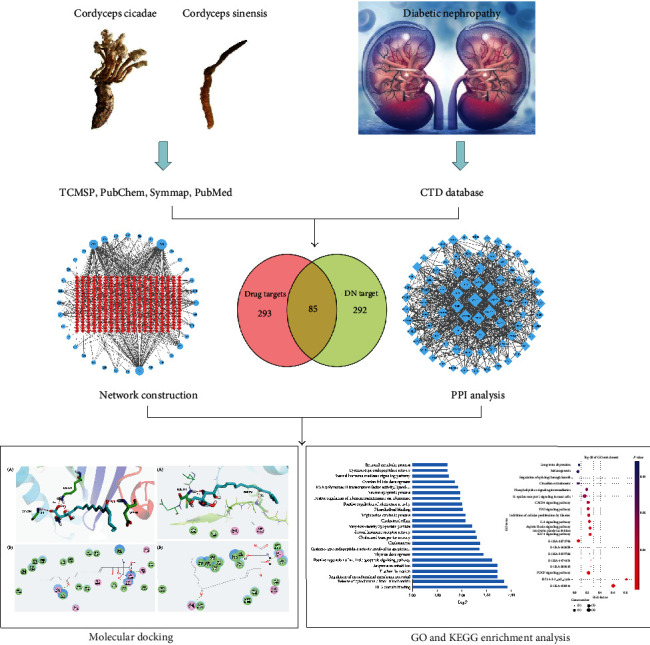
Network pharmacology workflow of cordyceps cicadae and diabetic nephropathy.

This study is aimed at exploring the therapeutic effects and mechanisms of cordyceps cicadae on DN by the network pharmacology approach. The workflow is shown in Figure 1.

## 2. Materials and Methods

### 2.1. Identification of Candidate Components

Most components are collected from the Traditional Chinese Medicine Systems Pharmacology Database and Analysis Platform (TCMSP) database (http://tcmspw.com/tcmsp.php). On account of that, cordyceps cicadae contains almost all the same active components of cordyceps sinensis; the keywords are “Cordyceps sinensis” and “cordyceps cicadae” so that the eligible active components and corresponding targets are collected. Symmap database (http://www.symmap.org/) was used to search all the active components and targets with the keywords of “Cordyceps sinensis” and “cordyceps cicadae.” The compounds and corresponding targets with OB ≥ 30% were screened out, and the repetitive compounds were removed. Moreover, PubMed (https://pubmed.ncbi.nlm.nih.gov) was used to search the literature for further information. Finally, the candidate active compounds were identified.

### 2.2. Prediction Targets of Candidate Active Compounds

The targets were obtained from four aspects: (1) PubChem database (http://pubchem.ncbi.nlm.nih.gov), (2) TCMSP database, (3) Symmap database, and (4) PubMed database.

### 2.3. Prediction Targets of Diabetic Nephropathy

With “diabetic nephropathy” as keywords, the CTD database was used (http://ctdbase.org/about/) to search genes related to diabetic nephropathy.

### 2.4. Network Construction

The active component target network of cordyceps cicadae was constructed by using the software Cytoscape 3.6.0 to analyze the association between components and targets, and the degree between candidate compounds and targets was analyzed.

### 2.5. Protein-Protein Interaction (PPI) Network Construction

The intersection targets of candidate compound and diabetic nephropathy were obtained by using the omicshare website (http://www.omicshare.com/). The intersection targets of candidate compound and diabetic nephropathy were imported into the STRING database (https://string-db.org/) to obtain protein interaction information. The condition was limited to “Homo sapiens.” The confidence score with correlation degree was set to ≥0.700. The PPI data was imported into the software Cytoscape 3.6.0 to construct the network diagram of intersection target protein interaction.

### 2.6. GO and KEGG Enrichment Analysis for Targets

In order to further analyze the function of the selected targets and their role in the signal pathway, the intersection targets of the active components and diabetic nephropathy were uploaded to the DAVID database for biological process enrichment analysis and pathway enrichment analysis.

### 2.7. Prediction of Binding Modes between Myriocin and Candidate Two Target Proteins

Docking studies were finished by Discovery Studio 3.5 to explore the predicted binding modes of myriocin in AKT1 (PDB code: 3OCB) and MAPK8 (PDB code: 4G1W), respectively, which were downloaded from RCSB Protein Data Bank (http://www.pdb.org/). The proteins were prepared using the *Prepare Protein* protocol in DS3.5, to remove all crystallographic water, add hydrogen atoms, repair broken chains, and add CHARMm force field. A sphere binding site was generated using the define site tool in DS3.5. Before docking, the small molecule myriocin was prepared using the *Prepare or Filter Ligands* protocol in DS3.5. The CDOCKER protocol in DS3.5 was used to perform the molecular docking. The images were created by PyMOL.

## 3. Results

### 3.1. Screening of Bioactive Compounds from Cordyceps Cicadae

38 compounds of cordyceps sinensis were found by TCMSP database. 31 compounds of cordyceps sinensis were screened by Symmap database with OB ≥ 30%. 12 active compounds of cordyceps cicadae were found by literature. A total of 61 candidate compounds were found. According to these methods, 378 targets related to candidate compounds were found (Table 1).

### 3.2. Targets and Active Compounds of Cordyceps Cicadae Network Construction

**Figure 2:**
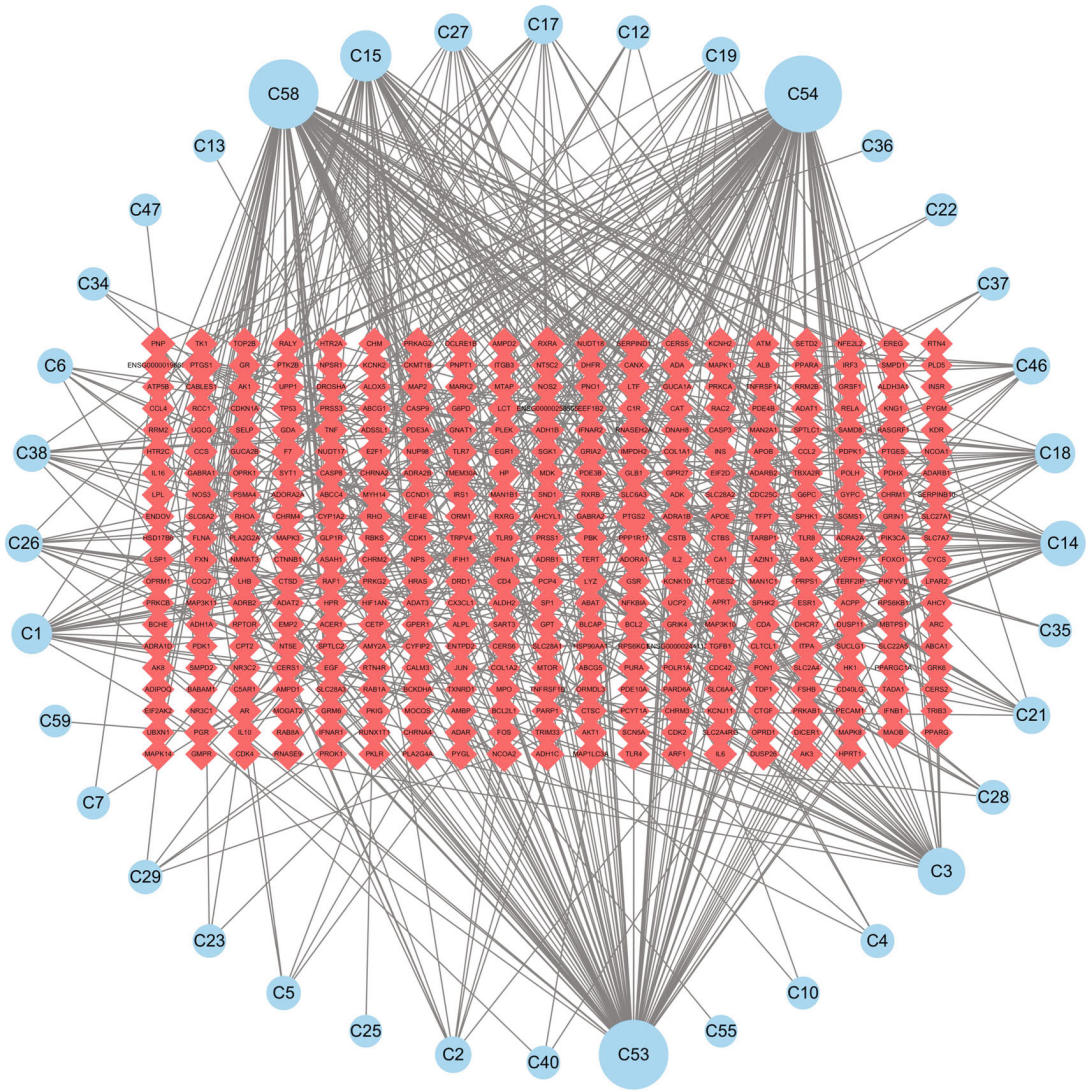
The active components and target network of cordyceps cicadae. The blue circle represents the component of cordyceps cicadae; the red diamond represents the target; the size of the node represents the size of the node degree.

Targets related to the candidate compounds were obtained by using the above methods, the components and targets were imported into the Cytoscape 3.6.0 software, and the network of active compounds and targets of cordyceps cicadae was obtained (shown in Figure 2). A total of 61 compounds were screened out, of which 25 compounds were not found corresponding targets in the database. Therefore, Figure 2 only showed the interaction network between 36 compounds and their related targets. The blue circle represented the candidate compounds, and the red diamond represented the targets. The more edges connecting to the node were, the higher the degree value of the node was. In this network, guanosine (C54) had 107 targets, myriocin (C58) had 89 targets, and inosine (C53) had 89 targets. Guanosine and inosine were the common components of cordyceps sinensis and cordyceps cicadae, and myriocin was the unique component of cordyceps cicadae. These multitarget compounds might be the core components of cordyceps cicadae. PTGS2 (prostaglandin endoperoxidase synthase 2) and PTGS1 (prostaglandin endoperoxidase synthase 1) were the targets with high degree value in the network, respectively. The targets with higher degree value might be the key targets for the efficacy of cordyceps sinensis and cordyceps cicadae.

### 3.3. Target-DN PPI Network

**Figure 3:**
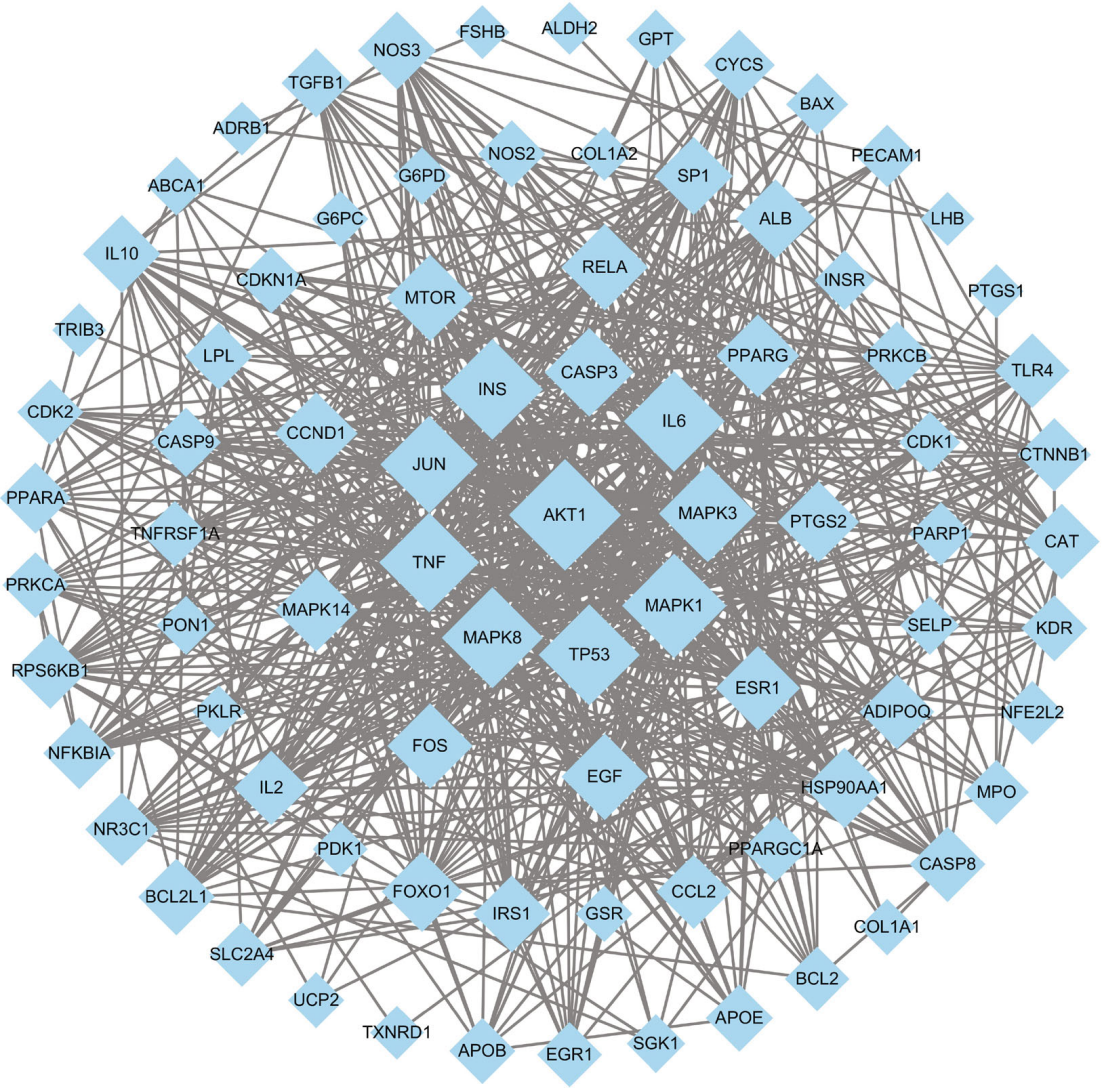
Target-diabetic nephropathy protein-protein interaction (PPI) network. AKT1, MAPK8, TP53, INS, MAPK1, IL6, TNF, JUN, MAPK3, and CASP3 were the targets with higher degree (>30).

377 genes related to DN were obtained in the CTD database with the inference score > 40. The 378 targets of compounds were intersected with 377 targets related to DN, and 85 important targets were obtained. These 85 targets were uploaded to the STRING database. The PPI data was obtained by setting the confidence level to 0.7, and then, the PPI data was imported into the Cytoscape 3.6.0 software to construct the network of protein target interaction (shown in Figure 3). Figure 3 shows a network of 85 interacting proteins with 1466 edges. The average degree of nodes is 34.5. AKT1, MAPK8, TP53, INS, MAPK1, IL6, TNF, JUN, MAPK3, and CASP3 were the targets with higher degree (>30).

### 3.4. Enrichment Analysis by GO and KEGG

**Figure 4:**
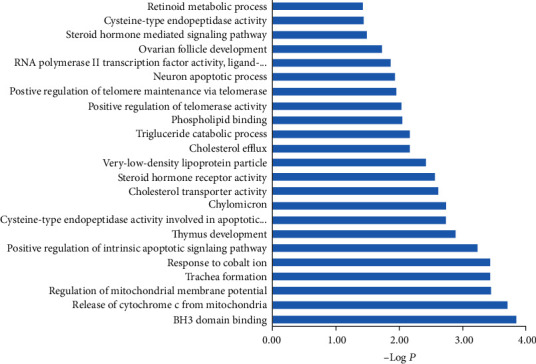
GO enrichment analysis of targets. The *y*-axis represents the biological process of the top 23 functional enrichment, and the *x*-axis represents the negative logarithm of *P* value.

**Figure 5:**
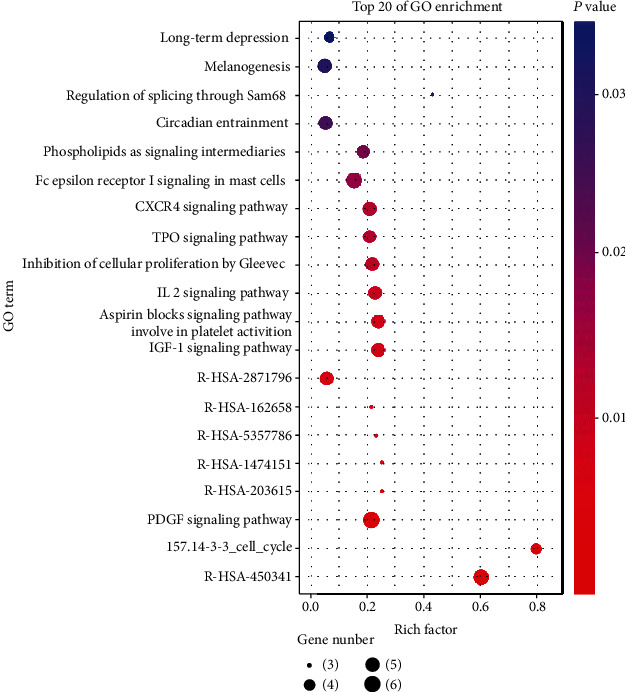
Top 20 of pathway enrichment. The circle size stands for gene numbers, the red color and higher rich factor indicate greater enrichment of pathways.

85 intersection targets were imported into the DAVID database for GO enrichment analysis and pathway enrichment analysis. The GO enrichment analysis is shown in Figure 4. The results of GO enrichment analysis showed that the key intersection targets were mainly concentrated in BH3 domain binding, release of cytochrome c from mitochondria, regulation of mitochondrial membrane potential, and other processes. The results of pathway enrichment analysis are shown in Figure 5. Rich factor represented the ratio of the number of genes in the pathway of differentially expressed genes to the total number of target genes in the pathway of all genes. The larger the rich factor was, the greater the degree of enrichment was. The key targets of this study were mainly enriched in the R-HSA-450341 signaling pathway, 157.14-3-3 cell cycle signaling pathway, and PDGF signaling pathway.

### 3.5. Molecular Docking

**Figure 6:**
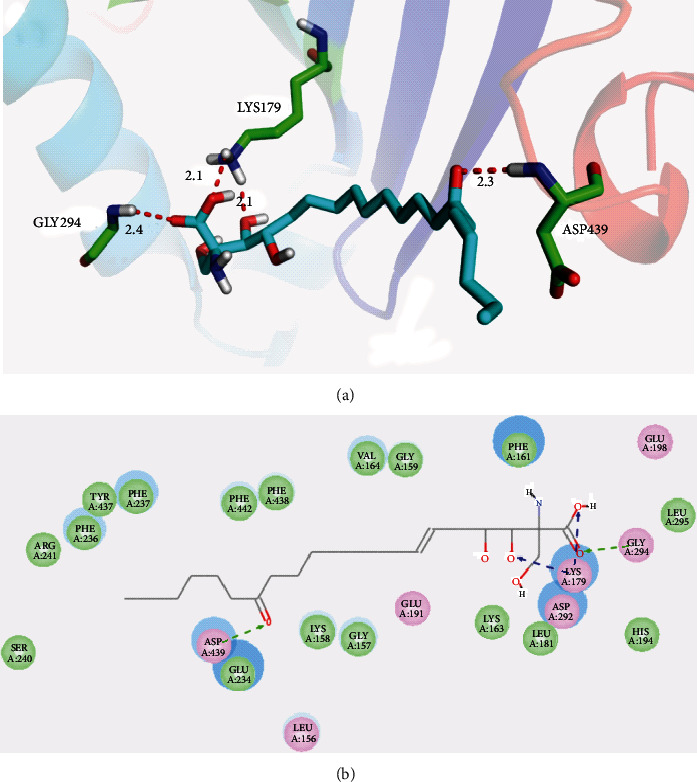
(a) Proposed binding mode of myriocin with AKT1 (PDB code: 3OCB). (b) 2D presentation of interaction between myriocin and AKT1.

**Figure 7:**
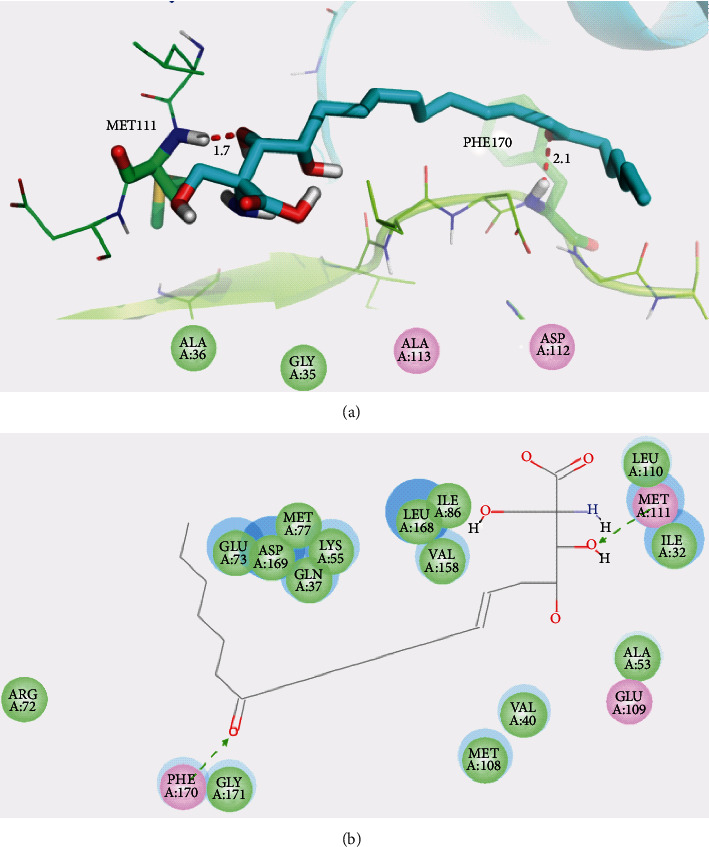
(a) Proposed binding mode of myriocin with MAPK8 (PDB code: 4G1W). (b) 2D presentation of interaction between myriocin and MAPK8.

Molecular docking studies were carried out to investigate the binding modes of myriocin with ATK1 and MAPK8. As shown in Figure 6(a), myriocin bound to AKT1 with four key hydrogen bonds. The carboxylic acid group of myriocin formed two hydrogen bonds with side chain of LYS179 (length: 2.1 Å) and backbone of GLY294 (length: 2.4 Å), respectively. One of hydroxyl formed one hydrogen bond with side chain of LYS179 (length: 2.1 Å). An additional hydrogen bond (length: 2.1 Å) was formed between carbonyl of myriocin and backbone of ASP439. Similarly, myriocin was docked into the binding pocket of MAKP8 through two hydrogen bonds with MET111 (length: 1.7 Å) and PHE170 (length: 2.1 Å), shown in Figure 7(a). Figures 6(b) and 7(b) show the interactions between myriocin and ATK1 and MAPK8, respectively, in 2D diagram.

## 4. Discussion

Diabetic nephropathy is one of the most frequent microvascular complications in diabetic patients and is the leading cause of end-stage renal disease all over the world [12]. In recent decades, many studies on DN have been carried out, and some results have been achieved. However, no better treatment drugs or measures have been produced so far. In recent years, Chinese traditional medicine in the treatment of DKD has also carried out a lot of researches and has made achievements. Wang et al.'s study has shown that cordyceps sinensis can protect renal tubules and promote renal tubules repair in animal models of drug-induced acute renal injury, thus improving renal function [13]. Researches showed that cordyceps cicadae could alleviate glomerulosclerosis and improve the chronic renal failure [14–16].

This study systematically studied the potential mechanism of cordyceps cicadae for the treatment of DN by the method of network pharmacology and molecular docking analysis and analyzed and constructed the “active ingredient-target-disease” network diagram of cordyceps cicadae for the treatment of DN. 36 main active ingredients of cordyceps cicadae and 85 common targets of DN, including myriocin, guanosine, and adenosine, were screened out through the database search and screening. Besides, key targets such as AKT1, MAPK8, MAPK1, TP53, IL6, and TNF were screened out. Molecular docking studies showed the binding modes of myriocin with ATK1 and MAPK8. AKT is a serine threonine kinase, a central regulator of cell growth, proliferation, survival, and metabolism. Podocyte injury is a predictive indicator of DN. It has been reported that can inhibit podocyte autophagy and aggravate its apoptosis through the AKT pathway [17]. IL-6 is a proinflammatory factor, and one of the important factors in the occurrence of DN is inflammation. DN patients showed a higher level of IL-6, which positively correlated with the extent of proteinuria [18]. IL-6 seemed to be a good biomarker of chronic kidney injury; the signal transduction inflammatory response it participated in was critical to the progression of DN. Study suggests that these IL-6 responses were mediated through a gp130-STAT3-dependent mechanism [19]. TP53 is also called TRIAP1. Under high-glucose conditions, TRIAP1 is a small conserved protein containing 76 amino acids. It is induced by TP53 under low-level genotoxic stress and helps reduce cell death. It has been reported that TRIAP1 interacts with heat shock protein 70 (HSP70) to regulate the apoptosis pathway [20, 21]. Studies have shown that TRIAP1 is a direct target of miR-770-5p. The expression of miR-770-5p in podocytes is upregulated under high-glucose conditions, and the downregulation of miR-770-5p by targeting TRIAP1 can eliminate podocytes induced by high glucose and inhibit cell apoptosis, which further proves that TP53 has a potential role in the occurrence and development of DN [22]. In a diabetic environment, the increasing production of glycation end products activates NF-*κ*B, MAPK, and other signaling pathways, which in turn mediates the activation of TGF-*β* signaling pathways and promotes the synthesis and deposition of extracellular matrix [23]. MAPK8 is related to lipid metabolism [24], and lipid metabolism disorder is an important risk factor for the occurrence of DN. GO enrichment analysis results showed that the key targets were mainly enriched in BH3 domain binding, release of cytochrome c from mitochondria, and regulation of mitochondrial membrane potential biological processes. It was reported in the literature that the BH3 domain was involved in the process of cell apoptosis and autophagy [25]. Mitochondria also play a very important role in the process of cell apoptosis [26]. The results of pathway enrichment indicated that R-HSA-450341, 157.14-3-3 cell cycle, and PDGF signal pathways may be related to the treatment of DN by cordyceps sinensis. Among the active compounds of cordyceps cicadae, the candidate compounds that may be related to these targets include myriocin, *β*-sitosterol, guanosine, arachidonic acid, inosine, berberine, cinnamaldehyde, linoleyl acetate, uralene, linoleic acid, and deoxyandrographolide. Myriocin (ISP-1, thermozymocidin), an atypical amino acid, was isolated from the culture broth, mycelia, and sporoderm-broken spore powders of cordyceps cicadae [27]. The molecular docking studies showed that myriocin bound to AKT1 with four key hydrogen bonds as well as to MAKP8 through two hydrogen bonds. The results revealed that myriocin, one active compound of cordyceps cicadae, may act on AKT1 and MAPK8. However, the main active compounds of cordyceps cicadae such as myriocin that may act on the DN targets require further experimental verifications and explorations. The interaction of active compounds from cordyceps cicadae needs further research. In order to verify the effectiveness of network pharmacological screening of active compounds and targets, we will further use molecular biology methods to investigate the effects and molecular mechanism of candidate compounds on targets and their effects on DN. These potential mechanisms for the treatment of DN by cordyceps cicadae will provide good ideas and directions for the next step of experimental verification and innovative drug development.

In conclusion, our study predicted target proteins related to candidate compounds of cordyceps cicadae by network pharmacology and verified by molecular docking. Since traditional Chinese medicine contains a variety of active compounds, they can act on multiple targets and signal pathways at the same time and produce a synergistic effect, which can be an effective method for the treatment of DN. Moreover, we can find particularly active compounds of traditional Chinese medicine. It is possible to develop innovative drugs for the treatment of DN diseases and to break through the difficult problems in the treatment of DN.

## Figures and Tables

**Table 1:** Candidate compounds of cordyceps cicadae.

No.	Name of compounds	Number of targets	No.	Name of compounds	Number of targets
C1	Oleic acid	24	C32	Cerevisterol	0
C2	EIC	15	C33	Cholesteryl palmitate	0
C3	Arachidonic acid	38	C34	(2R,3S,5S)-5-(6-Aminopurin-9-yl)-2-(hydroxymethyl)oxolan-3-ol	3
C4	Linoleyl acetate	4	C35	(2R,3R,4S)-2-(6-Aminopurin-9-yl)-4-(hydroxymethyl)oxolan-3-ol	3
C5	Vitamin C	6	C36	Cordylagenin	1
C6	Uracil	13	C37	CLR	4
C7	Adenine	4	C38	Cinnamaldehyde	12
C8	Styrone	0	C39	Ignavine	0
C9	20-Hexadecanoylingenol	0	C40	Deoxyandrographolide	3
C10	Vitamin G	2	C41	Karakoline	0
C11	D-Mannoheptulose	0	C42	Isotalatizidine	0
C12	Ergosterol	5	C43	Neokadsuranic acid A	0
C13	MTL	2	C44	2,7-Dideacetyl-2,7-dibenzoyl-taxayunnanine F	0
C14	Beta-sitosterol	38	C45	3-Acetylaconitine	0
C15	Caffeine	54	C46	Berberine	12
C16	TGL	0	C47	Neokadsuranic acid C	1
C17	Nicotinic acid	17	C48	Hypaconitine	0
C18	GLB	34	C49	Deoxyaconitine	0
C19	Uralene	15	C50	Adenosine	0
C20	TRE	0	C51	N-(2-Hydroxyethyl)adenosine	0
C21	GUP	23	C52	1-[(2R,3R,4S,5S)-3,4-Dihydroxy-5-(hydroxymethyl)oxolan-2-yl]pyrimidine-2,4-dione	0
C22	Uridine	2	C53	Inosine	89
C23	Thiamine	4	C54	Guanosine	107
C24	Peroxyergosterol	0	C55	GUN	1
C25	Galactomannan	1	C56	Beauverin	0
C26	Palmitic acid	17	C57	N-(4-Aminobenzoyl)-L-glutamic acid	0
C27	Linoleic	14	C58	Myriocin	89
C28	NCA	7	C59	Ergosta-4,6,8(14),22-tetraene-3-one	1
C29	Stearic acid	7	C60	Hyaluronic acid	0
C30	LFA	0	C61	5,6-Epoxyergosta-7,22-dien-3-ol	0
C31	Isoergotamine	0			

## Data Availability

The data used to support the findings of this study are available from the corresponding author upon request.
